# Supplementary figures and images for: Foliar spraying of indoleacetic acid (IAA) enhances the phytostabilization of Pb in naturally tolerant ryegrass by limiting the root-to-shoot transfer of Pb and improving plant growth
Source: PeerJ. 2023 Dec 15;11:e16560. doi: 10.7717/peerj.16560 (PMC10726742; doi:10.7717/peerj.16560)

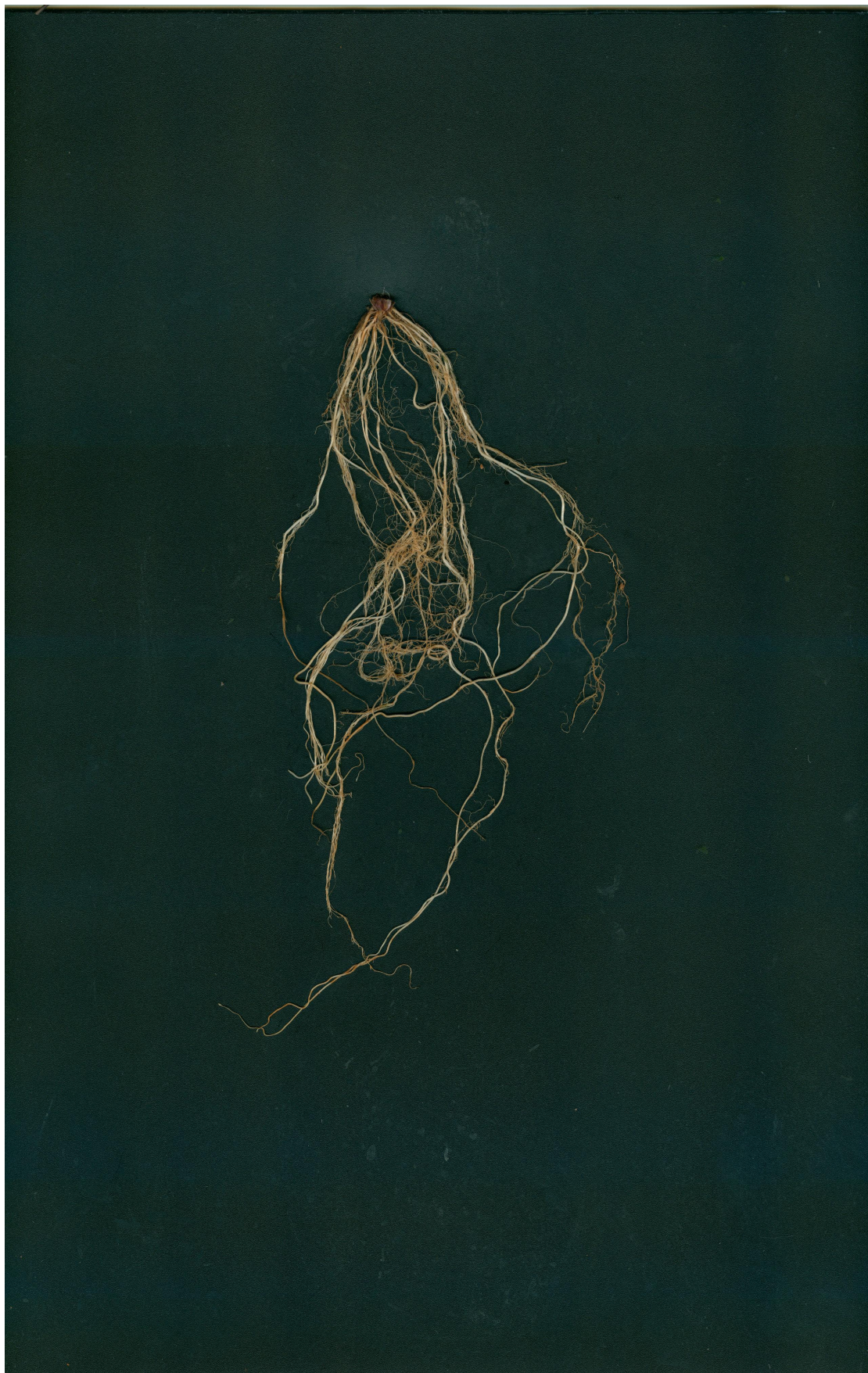

IAA+250mg/kg Pb

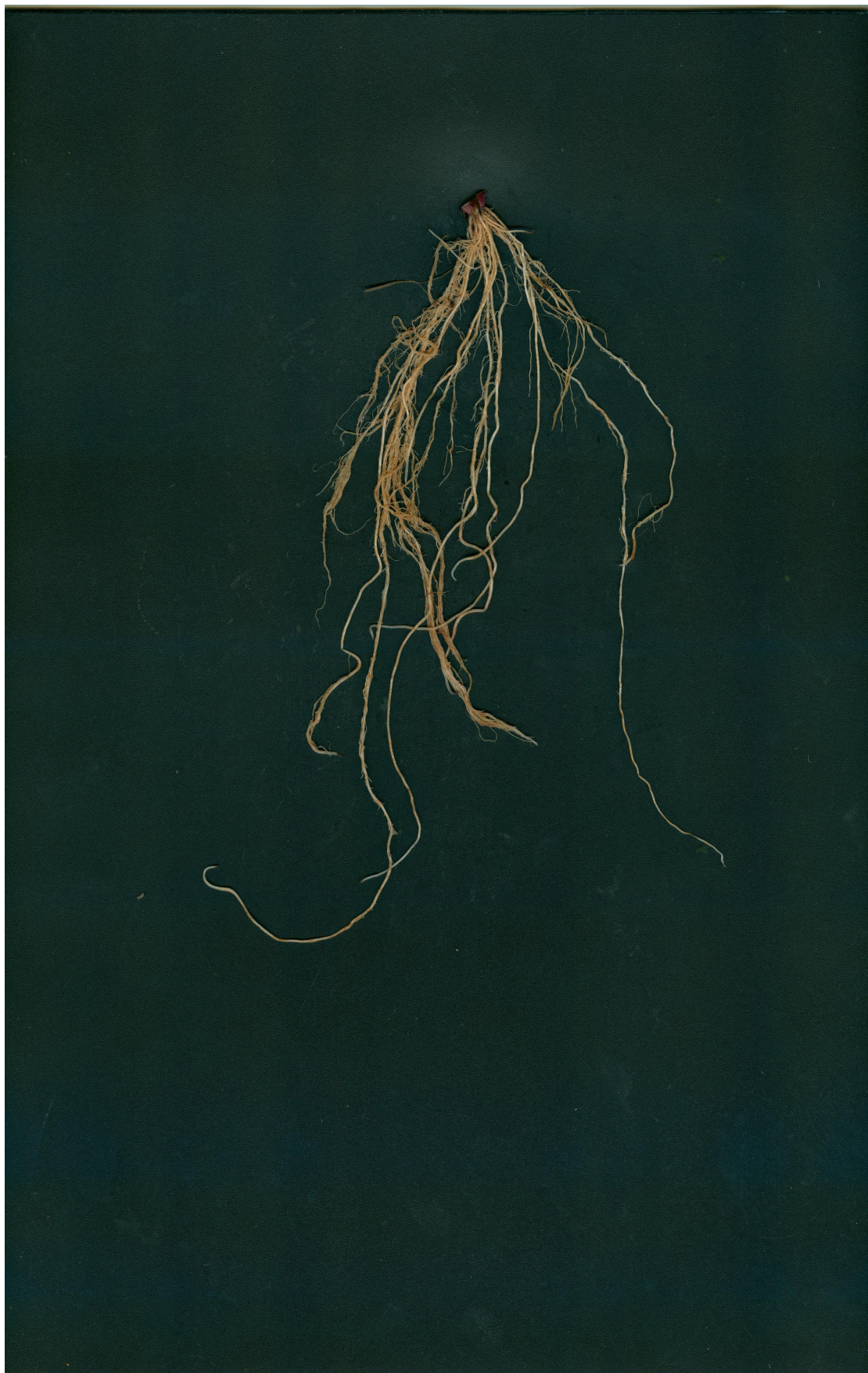

IAA+1000mg/kg Pb

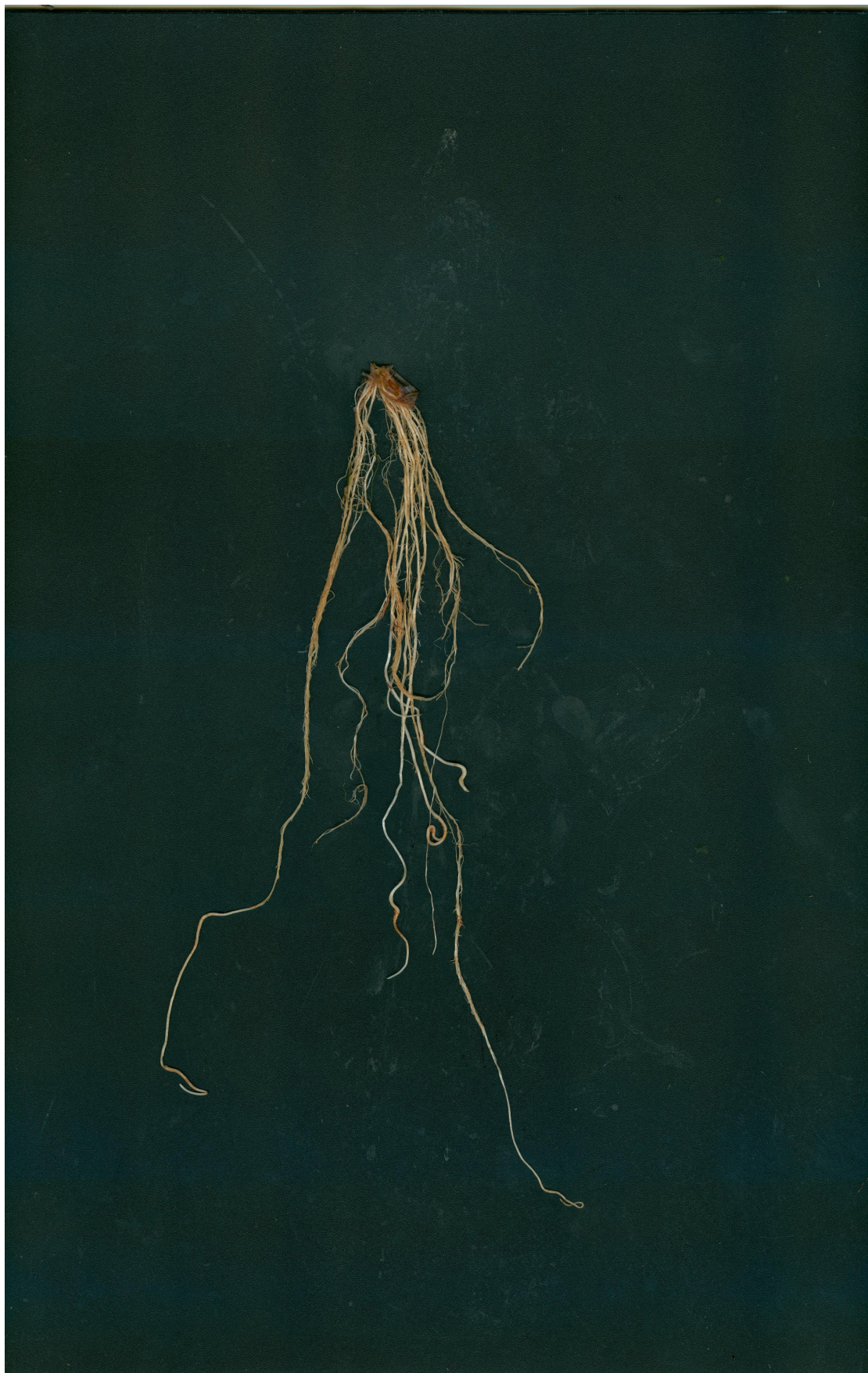

250mg/kg Pb

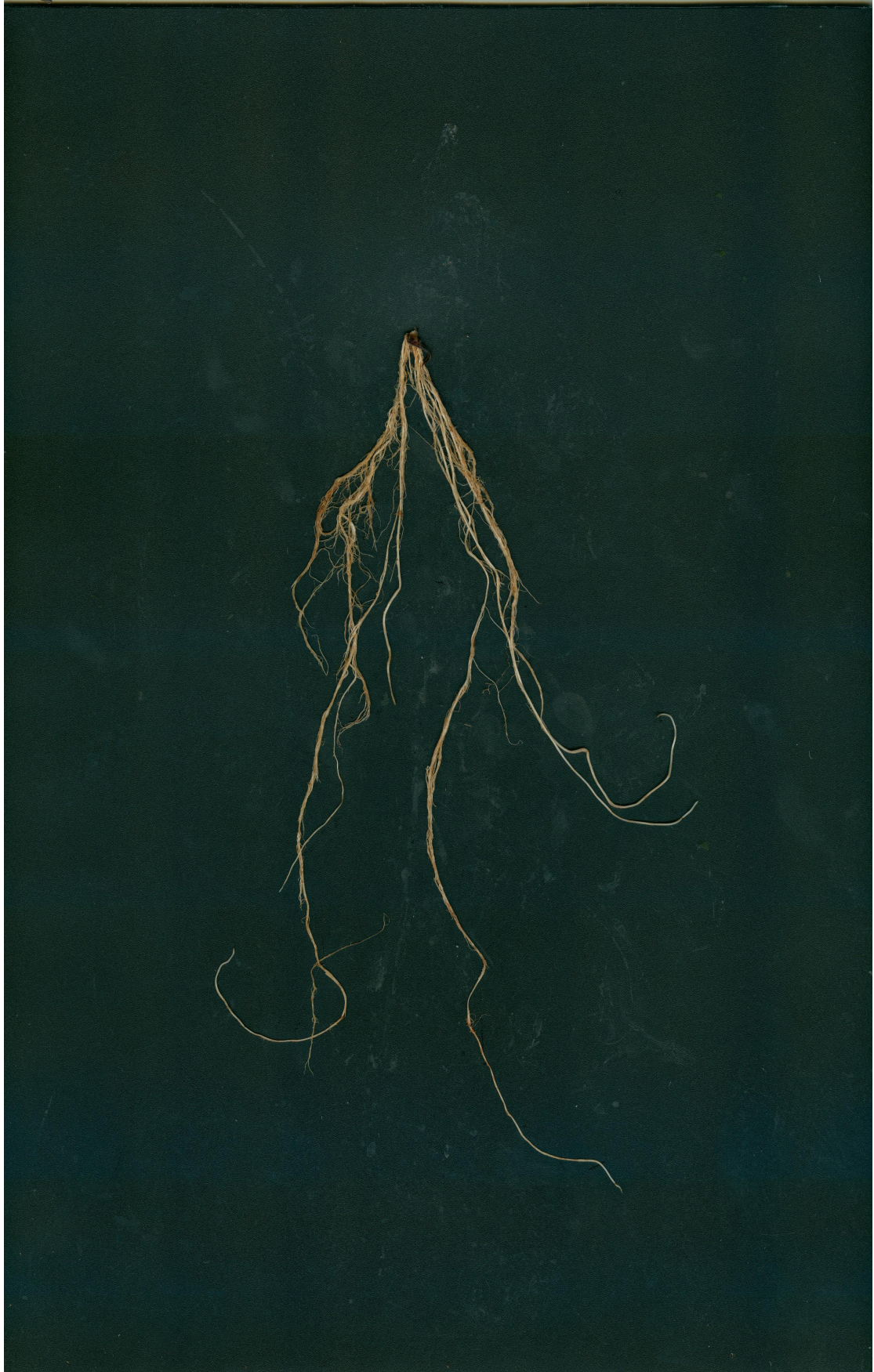

1000mg/kg Pb

Supplement: Supplemental Information 2 — Photo credit: Chengqiang Zhu. [file peerj-11-16560-s002.pdf]

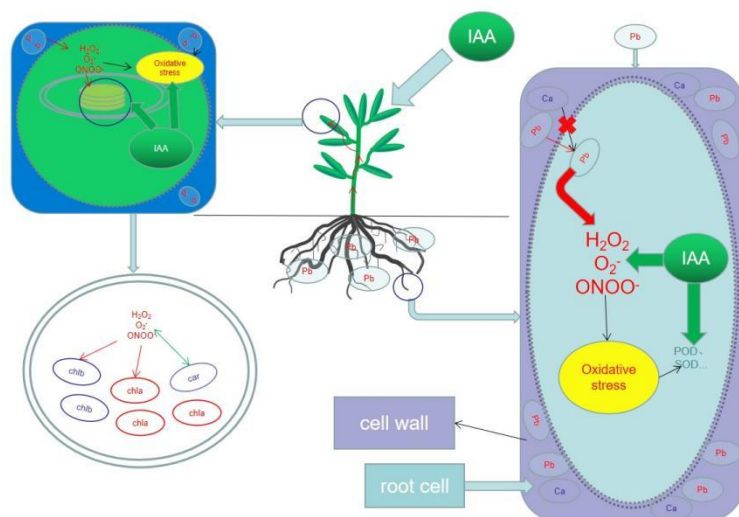

Supplement: Supplemental Information 3 [file peerj-11-16560-s003.pdf]
